# Automatic Prediction of Protein 3D Structures by Probabilistic Multi-template Homology Modeling

**DOI:** 10.1371/journal.pcbi.1004343

**Published:** 2015-10-23

**Authors:** Armin Meier, Johannes Söding

**Affiliations:** 1 Quantitative and Computational Biology, Max Planck Institute for Biophysical Chemistry, Göttingen, Germany; 2 Gene Center, Ludwig-Maximilians-Universität München Munich, Munich, Germany; Tel Aviv University, ISRAEL

## Abstract

Homology modeling predicts the 3D structure of a query protein based on the sequence alignment with one or more template proteins of known structure. Its great importance for biological research is owed to its speed, simplicity, reliability and wide applicability, covering more than half of the residues in protein sequence space. Although multiple templates have been shown to generally increase model quality over single templates, the information from multiple templates has so far been combined using empirically motivated, heuristic approaches.

We present here a rigorous statistical framework for multi-template homology modeling. First, we find that the query proteins’ atomic distance restraints can be accurately described by two-component Gaussian mixtures. This insight allowed us to apply the standard laws of probability theory to combine restraints from multiple templates. Second, we derive theoretically optimal weights to correct for the redundancy among related templates. Third, a heuristic template selection strategy is proposed.

We improve the average GDT-ha model quality score by 11% over single template modeling and by 6.5% over a conventional multi-template approach on a set of 1000 query proteins. Robustness with respect to wrong constraints is likewise improved. We have integrated our multi-template modeling approach with the popular MODELLER homology modeling software in our free HHpred server http://toolkit.tuebingen.mpg.de/hhpred and also offer open source software for running MODELLER with the new restraints at https://bitbucket.org/soedinglab/hh-suite.

## Author Summary

Since a protein’s function is largely determined by its structure, predicting a protein’s structure from its amino acid sequence can be very useful to understand its molecular functions and its role in biological pathways. By far the most widely used computational approach for protein structure prediction relies on detecting a homologous relationship with a protein of known structure and using this protein as a template to model the structure of the query protein on it. The basic concepts of this homology modelling approach have not changed during the last 20 years. In this study we extend the probabilistic formulation of homology modelling to the consistent treatment of multiple templates. Our new theoretical approach allowed us to improve the quality of homology models by 11% over a baseline single-template approach and by 6.5% over a multi-template approach.

This is a *PLOS Computational Biology* Methods paper.

## Introduction

Homology modeling is by far the most widely used computational approach to predict the 3D structures of proteins, and almost all protein structure prediction servers rely chiefly on homology modeling, as seen in the community-wide blind benchmark “Critical Assessment of Techniques for Protein Structure Prediction” (CASP) [1–3].

Homology modeling consists of four steps: (1) Finding homologous template proteins of known structure, (2) Selecting the best template or set of templates, (3) Optimizing the multiple sequence alignment (MSA) between query and template protein sequences, and (4) Building the homology model for the query sequence that resembles as closely as possible the structures of the templates, accommodating for deletions and insertions of query residues with respect to the template structures.

During the last 15 years, much progress has been made regarding the sequence-based steps 1 to 3. This is mainly owed to the development of more sensitive and accurate methods for sequence searching and alignment that compare sequence profiles or profile hidden Markov models (Hmms) with each other [4–6]. In contrast, improvements to the last step have been marginal. This is illustrated by the fact that, although a number of tools for protein homology modeling exist, to our knowledge all are older than 12 years (see [7, 8] for reviews). ModSeg/ENCAD [9] copies template coordinates and bridges gaps by short fragments that match the framework of the target structure. SWISS-MODEL [10] generates a core model by averaging template backbone atom positions. NEST [11] implements an artificial evolution algorithm where changes from the template structure such as substitutions, insertions and deletions are made one at a time, and each mutation is followed by an energy minimization. This process is repeated until the whole query is modeled.

These tools rely on of various heuristics. MODELLER [12], with 7500 citations clearly the most popular and according to two studies [7, 8] also the most successful homology modeling software to date, stands out by being based on a statistical approach to homology modeling. MODELLER is essentially unchanged at its core since its publication 22 years ago, while extensions such as refined energy functions [13] or loop modeling [14] have led to relatively minor improvements of its already excellent performance. We therefore believe Modeller’s success is owed to the consistent, statistical approach at its core.

**Fig 1:**
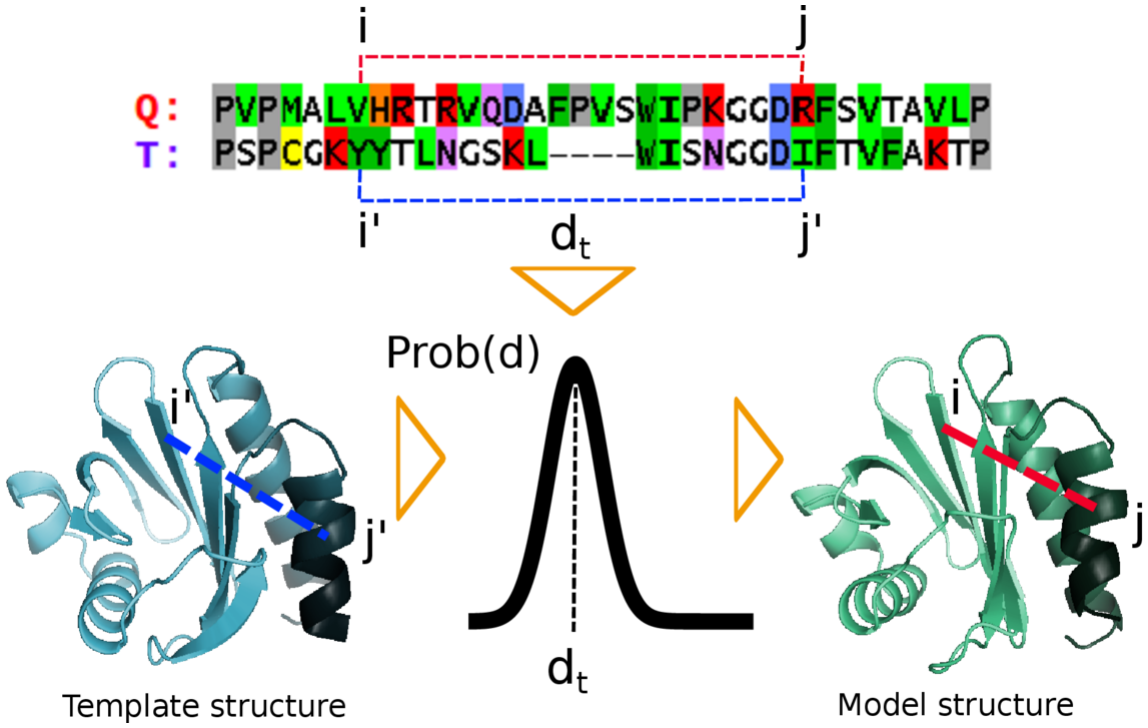
MODELLER’s statistical approach to homology modeling: The unknown distance *d* between two atoms in residues *i* and *j* of the query protein (Q) is described by a probability distribution Prob(*d*) that is peaked around the distance *d*_*t*_ between the corresponding atoms in residues *i*′ and *j*′ of the template protein (T). This distribution Prob(*d*) is a probabilistic distance restraint for the distance *d*. To model a protein, tens to hundreds of thousands of such distance restraints between pairs of atoms in the query protein are derived. The product of all these restraint functions, which is called the likelihood function in statistics, quantifies how well a model structure satisfies all restraints at the same time. Therefore, the model structure that maximises the likelihood function represents the best solution.

Modeller proceeds in two steps: (1) Derive from the MSA and template structures a list of restraints and (2) find the model structure that minimizes the restraint violations. Each restraint is a probability density function. The most important class of template-dependent restraints are the probability density functions for the spatial distances of pairs of atoms in the query protein. The true distance *d* will be distributed around the distance *d*_*t*_ of the equivalent atoms in the template structure, where equivalent residues are those that are aligned to each other (Fig 1). MODELLER assumes for simplicity a Gaussian distribution for *d*. Its mean equals *d*_*t*_ and its standard deviation is predicted based on the sequence similarity between query and template. The restraint minimization in the second step amounts to a maximum likelihood optimization, where the likelihood is approximated as the product over the density functions of the individual restraints. This factorisation of the likelihood assumes that the individual restraints represent information independent of each other, because in probability theory the joint probability of two random variables (*X* and *Y*) is the product of their probabilities, *p*(*X*, *Y*) = *p*(*X*) *p*(*Y*), if and only if they are independent of each other. Although the assumption of independence of restraints sounds rather drastic, the approximation turned out to work well in practice.

To aggregate the information from several templates, however, MODELLER does not multiply the density functions of all restraints as probability theory would suggest. Instead, it relies on an empirical observation that the distribution of the target distance informed by multiple template distances is multi-modal. Thus, MODELLER reverts to a heuristic approach and computes an additive mixture of the density functions, each derived from an individual template, to restrain a single target distance based on multiple templates.

Here, we develop a rigorous statistical treatment of multiple template homology modeling. We first show that the distance distributions for log(*d*) are very well described by two-component Gaussian mixture distributions. In contrast to MODELLER’s one-component densities, these two-component densities allow us to combine density functions by multiplication. Second, we derive an algorithm to compute weights that take the statistical dependence of the distance information from the templates into account. Third, we propose a heuristic scheme for template selection. We demonstrate that the new HHpred modeling pipeline and in particular the new constraints yield substantially improved model qualities.

## Materials and Methods

### Modeling distance restraints

Our approach to multi-template homology modeling is based on the statistical approach to homology modeling introduced by Modeller. Our software computes improved spatial restraints and calls the Modeller software, which then reads in the restraints and finds a structure that optimally satisfies these restraints. We briefly recall Modeller’s approach of homology modeling here.

#### Modeller’s maximum likelihood approach to homology modeling

Modeller proceeds in two steps to compute a model structure for a query sequence that is aligned to a set of templates with known structures. In the first step, it generates a list of hundreds of thousands of restraints for the distance between pairs of atoms in the query, based on the distance of corresponding atoms in the templates. E.g. if residue *i* of the query *q* is aligned to residue *i*′ of a template *t* and similarly *j* is aligned to *j*′, then the distance *d* between the C_*α*_ atoms of residues *i* and *j* in *q* will be restrained to be similar to the known distance *d*_*t*_ between the C_*α*_ atoms of residues *i*′ and *j*′ in *t* (Fig 1). In statistics, a restraint is described as a probability density function *p*(*d*), and in Modeller this distance restraint is modelled by a Gaussian function with mean *d*_*t*_. The standard deviation of the Gaussian describes the expected deviation of the distance *d* from *d*_*t*_. Distance restraints are generated for each pair of residues (*i*, *j*) for which aligned residues *i*′ and *j*′ exist and for various combinations of atom types, for which equivalent atoms exist in the aligned template residues, e.g. C_*α*_ − C_*α*_, N − O, C_*α*_ − C_*γ*_ etc.

In the second step, Modeller uses stochastic optimisation to find the model structure for the query sequence that maximises the likelihood. The likelihood is the probability of the data, i.e. the alignment and template structures, given the model structure. When a single template is used for modeling, Modeller approximates the likelihood as the product of the probability density functions over all restraints. Although this approximation corresponds to assuming the independence of all restraints, it has turned out to work well in practice.

Sali and Blundell [12] observed that the expected deviation *d* − *d*_*t*_ depended on (1) the fraction of identically aligned residues between the two sequences, (2) the average solvent accessibility of the two aligned residue pairs (*i*, *i*′) and (*j*, *j*′), (3) the average distance of *i*, *i*′, *j* and *j*′ from a gap, and (4) the distance *d*_*t*_. They modelled the standard deviation of the Gaussian restraint as functions of the four discretized variables. To fit these functions, they analysed a large set of structurally aligned, homologous proteins for which they measured the distances *d* = *d*_*ij*_ and *d*_*t*_ = *d*_*i*′*j*′_ between equivalent atoms in two pairs of structurally aligned residues, (*i*, *i*′) and (*j*, *j*′). Four different functions are trained, one for each of the following combinations of atom types: C_*α*_ − C_*α*_, N − O, side chain—main chain, side chain—side chain.

#### New distance restraints that account for alignment errors

Because the analysis in [12] relied on structurally alignable residue pairs in structure-based alignments, they were basically free of alignment errors and therefore the distance in the query was always similar to the distance in the template. In practice, the sequence alignment will contain errors and *i* and *i*′ (or *j* and *j*′) might not be homologous to each other. In this case, *d*_*t*_ does not contain information about *d* and may be vastly different. When the pairs of residues (*i*, *i*′) and (*j*, *j*′) are sampled from real sequence alignments, this may lead to a stark deviation of the distance distribution from a Gaussian.

**Fig 2:**
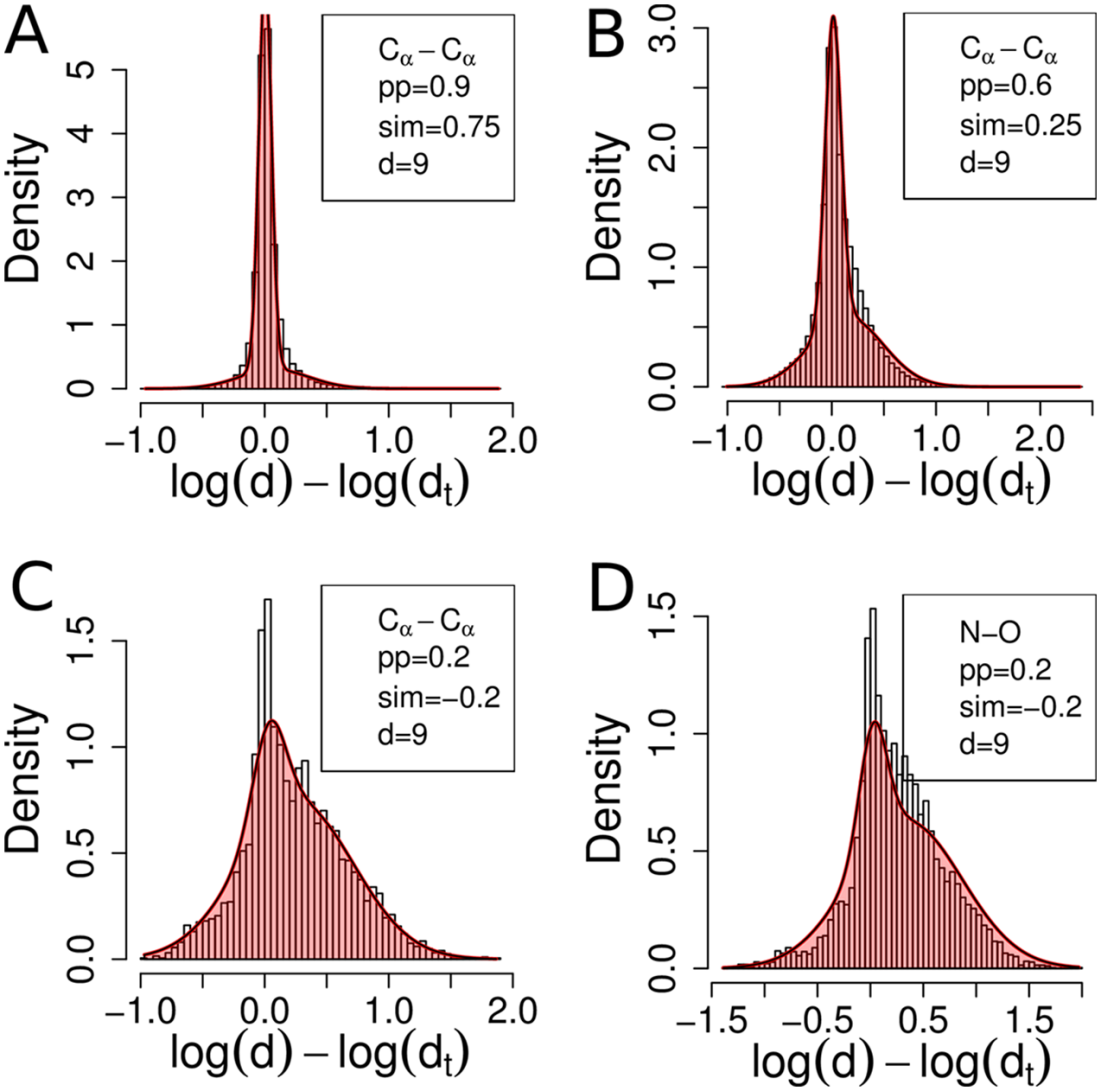
Empirical log distance distributions between pairs of atoms are well modelled by a two-component Gaussian mixture composed of a signal component and a background component. The background component originates from pairs of residues with an alignment error. The plots show the empirical distribution of log *d* − log *d*_*t*_ = log *d*_*ij*_ − log *d*_*i*′*j*′_ for thousands of sampled pairs of residues (*i*, *i*′), (*j*, *j*′) from real, error-containing pairwise sequence alignments generated with HHalign [15]. The two-component Gaussian mixture distribution predicted by the mixture density network in Fig 3B is plotted in red. From (**A**) to (**C**), the reliability of the alignments at (*i*, *i*′) and (*j*, *j*′) (as measured by pp and sim values) decreases. Consequently, the weight of the background component increases at the expense of the signal component. (**D**) Same as (C) but showing the distribution of N − O distances instead of C_*α*_ − C_*α*_ distances.

Fig 2(A)–2(C) shows distributions of log(*d*) − log(*d*_*t*_) for sets of residue pairs (*i*, *i*′) and (*j*, *j*′) sampled from alignments with successively lower quality. In Fig 2A only very reliable alignments have been sampled, with a posterior probability (pp) for (*i*, *i*′) and (*j*, *j*′) to be correctly aligned larger than 0.9 and with a sequence similarity (sim) above 0.75 bits per aligned pair. (See the Supporting Information for the definition of pp and sim.) Consequently, the empirical density distribution over log(*d*) − log(*d*_*t*_) has a single peak and is well fitted by a single Gaussian. However, when the alignment quality deteriorates, as shown in Fig 2B and 2C, a second component in the distribution manifests itself. It stems from residues (*i*, *i*′) and (*j*, *j*′) for which either (*i*, *i*′) or (*j*, *j*′) or both are not homologous. These data points thus contribute a background distribution that does not depend on the distance *d*_*t*_ in the template.

**Fig 3:**
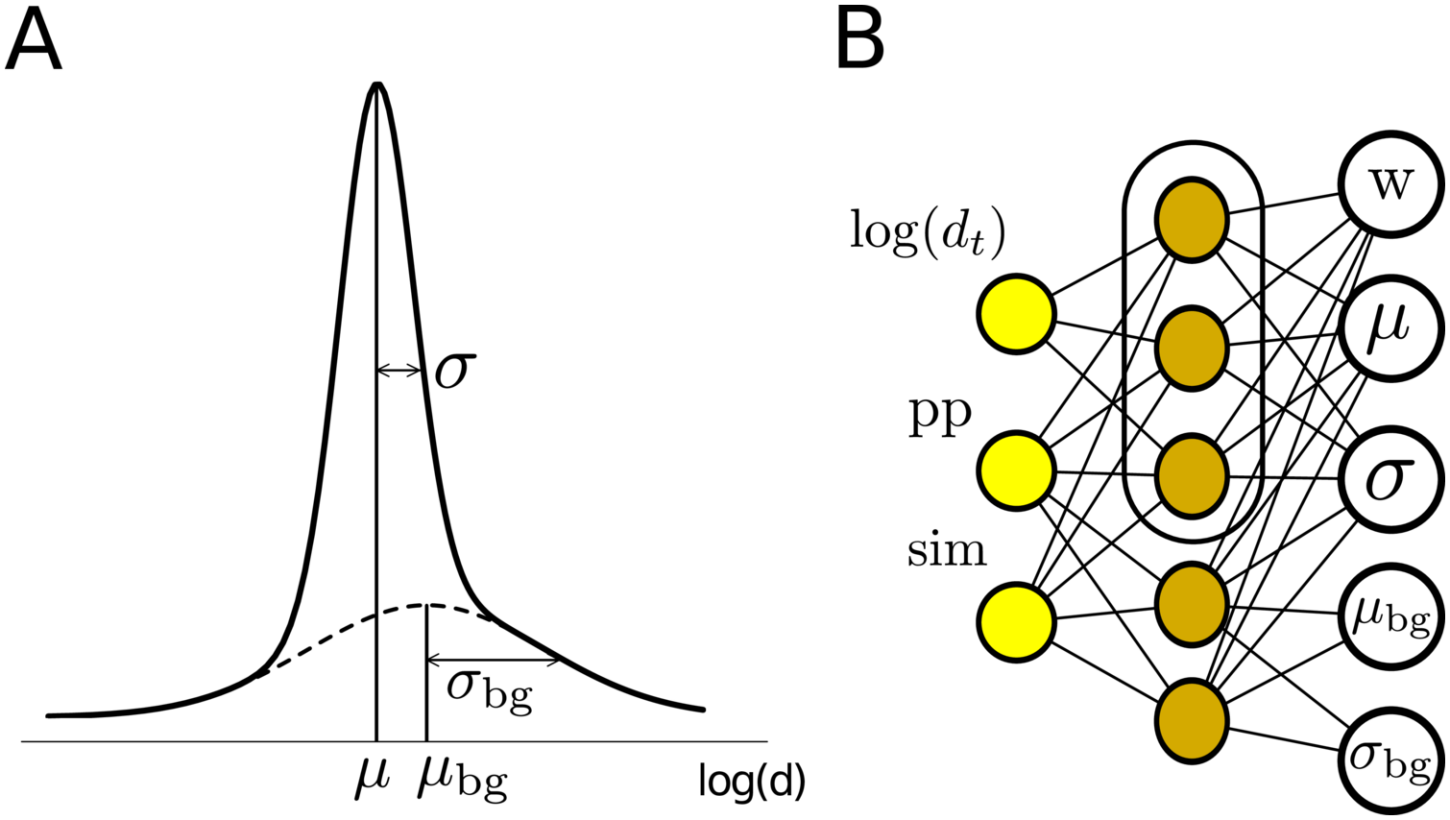
(**A**) Illustration of the two-component Gaussians mixture distribution in Eq (1). (**B**) Mixture density network to predict the parameters (*w*, *μ*, *σ*, *μ*_bg_, *σ*_bg_) of the Gaussian mixture distribution given the three variables *θ* = (log *d*_*t*_, pp, sim) (*d*_*t*_: distance in template, pp: posterior probability for both aligned residue pairs to be correctly aligned, sim: sequence similarity). Since the background component does not depend on *d*_*t*_, the nodes for *μ*_bg_ and *σ*_bg_ are only connected to the two lowest hidden nodes that are not connected to log *d*_*t*_.

These observations motivated us to model the restraint function *p*(log *d*∣ log *d*_*t*_, pp, sim) = *p*(log *d*∣*θ*) using a two-component Gaussian mixture distribution (see Fig 3A) whose means, standard deviations and mixture weight *w* depend on *θ* = (log *d*_*t*_, pp, sim) or *θ*′ = (pp, sim):

$$p\left(\log d|\theta \right)=\underset{\text{correctly}\;\text{aligned}}{\underset{︸}{w\left(\theta \right)N(\log d|\mu \left(\theta \right),\sigma^{2}\left(\theta \right))}}\;+\;\underset{\text{alignment}\;\text{errors}}{\underset{︸}{\left(1-w\left(\theta \right)\right)N\left(\log d|\mu_{\text{bg}}\left(\theta^{\prime }\right),\sigma_{\text{bg}}^{2}\left(\theta^{\prime }\right)\right)}}$$ (1)

The mixture weight *w*(*θ*) can be regarded as the probability that both (*i*, *i*′) and (*j*, *j*′) are correctly aligned. Locally unreliable alignments will lead to a stronger background component and hence to softer distance restraints. Note that, because distances cannot be negative, they are not well modelled by Gaussian distributions, whose left tail can penetrate into the negative domain. We therefore modeled the distribution of log *d* instead of *d*.

#### Mixture density networks

To predict the five parameters of the Gaussians mixture distribution in Eq (1), we trained four mixture density networks [16], one for each combination of atom types listed above. A mixture density network is a special kind of neural network that learns the optimum adaptive functions for predicting the parameters of a Gaussian mixture distribution. It is trained by maximizing the likelihood of a set of training data that consists of the input features together with the value log *d* whose distribution should be learned. We used the R package netlabR to implement a mixture density network with five hidden nodes as illustrated in Fig 3(B). As input features we used *θ* = (log *d*_*t*_, pp, sim). The local alignment quality pp(*i*, *j*) and the global BLOSUM62 sequence similarity sim are parsed from the output of HHalign in the hh-suite package [15], a widely used software for remote homology detection and sequence alignment (see Fig 8, green points). The set of three features was obtained by starting from a more redundant set of alignment features described in Table B in S1 Text and successively eliminating features whose omission did not significantly deteriorate the likelihood on the training set (in particular probability and raw score).

#### Combining restraints from multiple templates

**Fig 4:**
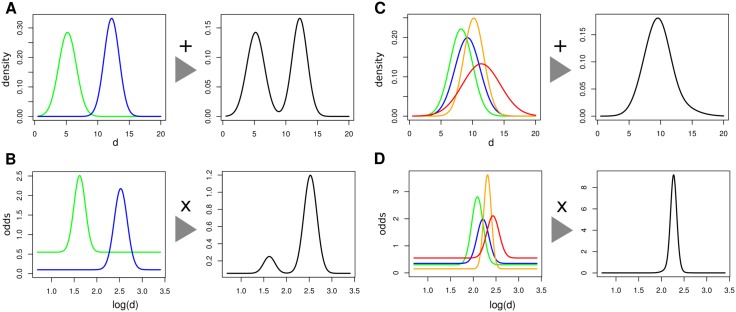
Comparison of how restraints from multiple templates are combined in Modeller (top row) and in our new approach (bottom row). **(A)** In Modeller, two restraints functions (green and blue) are additively mixed with mixing weights that have to be learned on a set of triples of aligned protein structures. **(B)** Our new restraints are multiplied instead of being added. The background component ensures that the restraint function becomes constant and the restraint thus becomes inactive (i.e. ignored) when the distance *d* is far from the distance in the template. **(C)** Modeller’s additive mixing leads to a total restraint function that is wider than any of the single-template restraints, not narrower as it should. **(D)** The multiplication of restraints functions according to probability theory leads to the desired behaviour of the total restraint function becoming more pointed with each restraint. Note that our new restraints are expressed as odds instead of densities (see also Eq 6).

When several templates cover residues *i* and *j* of the query, the restraints on the distance *d* of atoms in residues *i* and *j* from those templates have to be combined. Multiplying the restraint functions as probability theory would suggest (see below) would not work in Modeller’s case. When one of the restraints is wrong due to an alignment error, for instance, the restraint function of the incorrect restraint would severely distort the model structure, because the probability density of its single-component Gaussian falls off very fast for increasing distance from its mean, which effectively forbids any gross violation of the restraint. Therefore, Modeller resorts to a heuristic to estimate the probability density *p*(*d*∣*d*_1_, *d*_2_) resulting from the restraints of two templates *t*_1_, *t*_2_ with corresponding distances *d*_1_ and *d*_2_: It adds both probability densities *p*(*d*∣*d*_1_) and *p*(*d*∣*d*_2_) (Fig 4A) using some weights:

$$p\left(d|d_{1},d_{2},s_{1},s_{2}\right)\approx \alpha \left(s_{1}\right)\;p\left(d|d_{1}\right)+\alpha \left(s_{2}\right)\;p\left(d|d_{2}\right)\;.$$ (2)

Here *s*_1_ and *s*_2_ measure the average sequence similarity in the sequence neighbourhoods around the two pairs of aligned residues from *q* and *t*_1_ and from *q* and *t*_2_, respectively. The optimum functions *α*(*s*_1_), *α*(*s*_2_) were found by training on a large number of structurally aligned triplets of proteins *q*, *t*_1_, *t*_2_ [12].

This heuristic approach leads to undesirable behaviour, as illustrated in Fig 4A and 4C. According to elementary statistical principles, a restraint function for a distance *d* based on restraints from multiple templates should contain more information and be more sharply resolved than any single-template restraint function. However, the additive mixture density restraint in Eq (2) is wider, not narrower, than any single restraint.

The new two-component distance restraints allow us to apply the rules of probability to combine the information from the two templates. By Bayes’ theorem we obtain

$$p\left(d|d_{1},d_{2}\right)=\frac{p\left(d_{1},d_{2}|d\right)\;p\left(d\right)}{p(d_{1},d_{2})}\;.$$ (3)

If the information in the templates was approximately conditionally independent given *d*, i.e., *p*(*d*_1_, *d*_2_∣*d*) ≈ *p*(*d*_1_∣*d*) *p*(*d*_2_∣*d*) we would obtain

$$\frac{p(d|d_{1},d_{2})}{p(d)}\approx \frac{p(d_{1}|d)}{p(d_{1})}\;\frac{p(d_{2}|d)}{p(d_{2})}=\frac{p(d|d_{1})}{p(d)}\;\frac{p(d|d_{2})}{p(d)},$$ (4)

where Bayes’ theorem was applied to each factor in the second step.

In practice, the query and templates are related to each other through evolution along phylogenetic trees, and conditional independence cannot be assumed. We therefore approximate the dependence among the templates by weighting their odds ratios, with weights *w*_*k*_ ∈ [0, 1]. This method is analogous to weighting sequences according to their similarity with other sequences in a multiple sequence alignment in order to compute a sequence profile [17] or some other family-dependent features [18]. We will derive a method to determine optimal template-specific weights *w*_*k*_ in the following subsection. The previous formula can then be generalised to *K* templates, giving

$$\frac{p(d|d_{1},\ldots ,d_{K})}{p(d)}\approx \prod_{k=1}^{K}{\left(\frac{p(d|d_{k})}{p(d)}\right)}^{w_{k}}.$$ (5)

Here, *p*(*d*) is the probability independent of any template, i.e., the background distribution $N(d|\mu_{\text{bg}},\sigma_{\text{bg}}^{2})$. According to Eq (1), the restraint functions are now (for the sake of brevity we omit *θ* and *θ*′)

$$\frac{p(d|d_{k})}{p(d)}=\frac{\left(1-w\right)N\left(\log d|\mu_{\text{bg}},\sigma_{\text{bg}}^{2}\right)+wN\left(\log d|\mu ,\sigma^{2}\right)}{N(\log d|\mu_{\text{bg}},\sigma_{\text{bg}}^{2})}=1\;-w\;+w\frac{N(\log d|\mu ,\sigma^{2})}{N(\log d|\mu_{\text{bg}},\sigma_{\text{bg}}^{2})}\;.$$ (6)

Note that the ratio of the two Gaussians is again a Gaussian, because subtracting two quadratic functions of *d* again yields a quadratic function. Fig 4B and 4D illustrate how restraints from multiple templates are combined under our new statistical approach and that this leads to the expected desirable behaviour of the total restraint restraining more strongly than the one-component restraints.

Dividing by the background has two effects: first, it prevents the background to become dominant when the individual background components of all *P*(*d*∣*d*_*k*_) are multiplied. Second, the negative logarithm of Modeller’s distance restraint is quadratic in *d*, and hence unsatisfiable restraints can lead to extreme values during optimization. Dividing by the background avoids this quadratic increase because *P*(*d*∣*d*_*k*_)/*P*(*d*) has flat tails where it approaches a constant (1 − *w*). In cases of incorrect alignments with a wrong distance *d*_*t*_ in the template, the restraint will not disrupt the query’s model structure as *d* will be pulled away from *d*_*t*_ into the flat region of the restraint. Combining two component distance restraints as shown in Fig 4D thus reinforces consistent restraints while avoiding distortions from incorrect restraints.

#### Running Modeller with the new distance restraints

After having picked a set of templates, we run the Modeller (version 9.10) automodel.homcsr(0) command that generates a file with the list of restraints from the query-template alignment. We parse the list of restraints and replace each template-dependent distance constraint (which is either a Gaussian function for a single-template restraint or a Gaussian mixture for a multi-template restraint) with a set of our own distance restraints, one for each template. For this purpose, we added a restraint function that computes the logarithm of Eq (6) to Modeller. All template-independent restraints such as main chain and side chain dihedral angle restraints, bond lengths etc. are left unchanged. We run Modeller with the modified restraints list to generate a 3D model.

### Template weighting

#### Motivation

**Fig 5:**
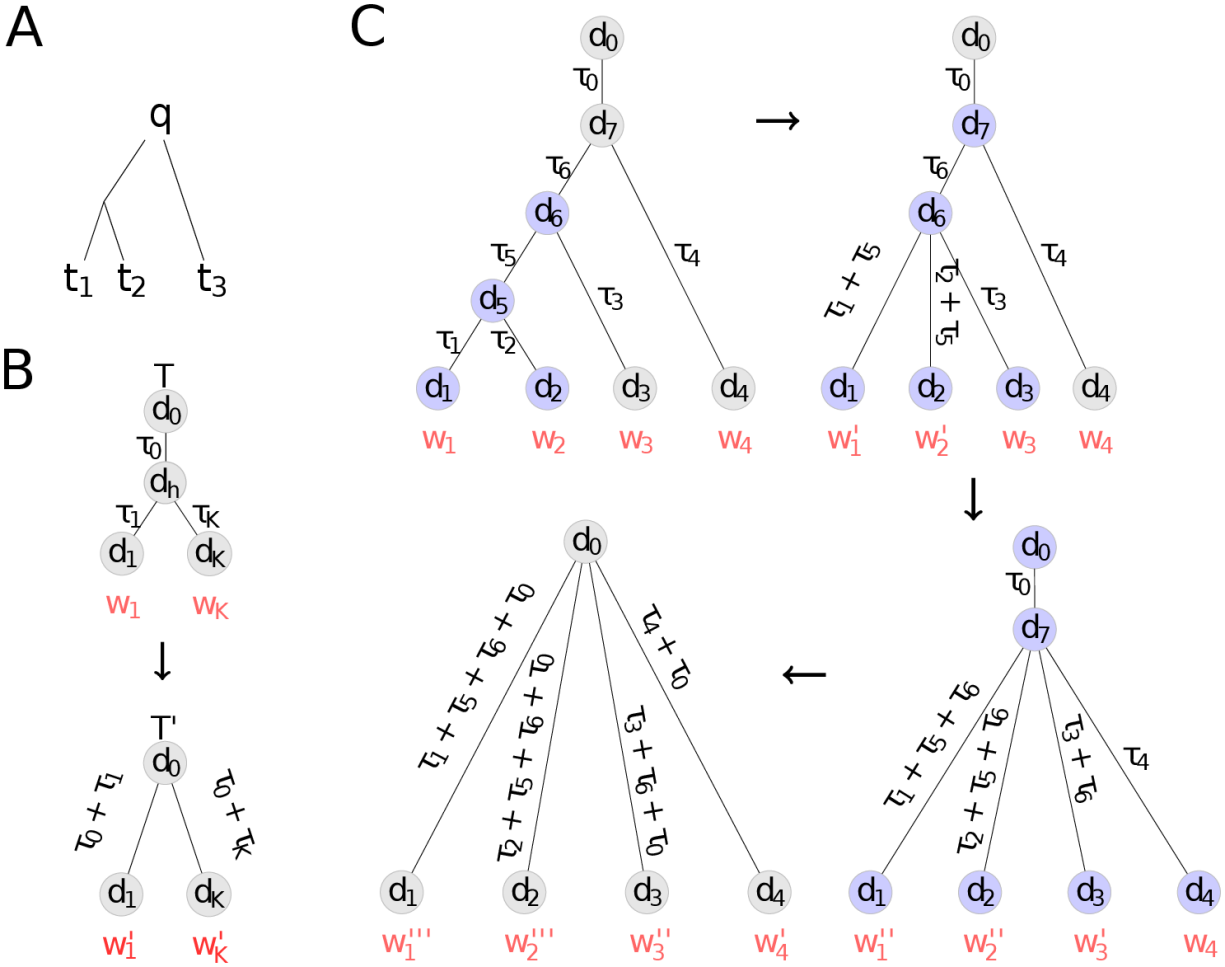
Iterative scheme for computing weights for templates by transforming the phylogenetic tree connecting them and the query protein into an equivalent tree with star-like topology with the query in the center. **(A)** Templates *t*_1_ and *t*_2_ are closely related and should be down-weighted with respect to *t*_3_. **(B)** Any tree T with a structure at an internal node with unknown distance *d*_*h*_ to which all templates are connected in a star-like topology (top) can be transformed into an equivalent tree $T^{\prime }$ (bottom) with star-like topology, where equivalence means that the restraint on the distance *d*_0_ of the top node is the same for both trees. *τ*_1_, … *τ*_*K*_ indicate evolutionary distances. **(C)** Iterative restructuring of a phylogenetic tree. In each step, the basic transformation from Fig 5B is applied to the subtree colored in blue. Weights and edge lengths get updated until all templates are directly connected to the query.

As a motivation for the template weighting scheme, consider the case shown in Fig 5A. Giving all three templates the same weight ignores the dependencies described by the tree [18]. Template *t*_3_ should get a weight of 1, since conditioned on *q* it is independent of the other two templates. But templates *t*_1_ and *t*_2_ should get weights clearly smaller than 1, since they do not contribute independent information to *d*. On the other hand, they are not identical and hence should receive a weight clearly larger than 0.5. But how do we compute the exact optimum weights *w*_*k*_ for templates 1, …, *K* given a phylogenetic tree with known edge lengths?

#### Iterative restructuring

We begin by rooting the phylogenetic tree at the query, and giving its leaf nodes initial weights of 1. By iteratively applying the elementary step in Fig 5B to subtrees, we can transform a tree with arbitrary topology into a tree with a star-like topology, as shown in Fig 5C. At each step, one inner node is removed and the procedure continues until all template leaves are directly connected to the query. At each step, we simply need to update the template weights to obtain the final weights *w*_*k*_ for the star-like tree. In the star-like tree which we finally obtain, all template distances *d*_*k*_ are conditionally independent, and hence we obtain for the odds ratio the result in Eq 5, using the final weights *w*_*k*_ from this iterative process.

#### Elementary step

For the elementary step, we will show that the upper (sub)tree T in Fig 5B yields exactly the same odds ratio for *d*_0_ as the transformed, star-topology tree $T^{\prime }$ below,

$$\frac{p(d_{0}|d_{1}\ldots d_{K},w_{1}\ldots ,w_{K},T)}{p(d_{0})}=\frac{p(d_{0}|d_{1}\ldots d_{K},w_{1}^{\prime }\ldots ,w_{K}^{\prime },T^{\prime })}{p(d_{0})},$$ (7)

if the new weights $w_{k}^{\prime }$ are chosen according to

$$w_{k}^{\prime }=\frac{1/\tau_{0}+1/\tau_{k}}{1/\tau_{0}+\sum_{l=1}^{K}w_{l}/\tau_{l}}\;w_{k}.$$ (8)

The updated weights are proportional to the old *w*_*k*_ with a proportionality factor approaching 1 for *τ*_0_ ≪ *τ*_*k*_. The sum of weights over all *K* templates is $\left(\sum_{k=1}^{K}w_{k}/\tau_{0}+\sum_{k=1}^{K}w_{k}/\tau_{k}\right)/\left(1/\tau_{0}+\sum_{k=1}^{K}w_{k}/\tau_{k}\right)$, which goes to 1 for *τ*_0_ ≫ max{*τ*_*k*_}, signifying that in this case the information in the templates is completely redundant.

To show that the odds ratio in Eq (7) is conserved when transforming the tree T into $T^{\prime }$ in Fig 5B, we integrate over the unknown, hidden distance *d*_*h*_,

$$p\left(d_{0}|d_{1}\ldots d_{K},w_{1}\ldots ,w_{K},T\right)=\int p\left(d_{0}|d_{h},w_{0}\right)p\left(d_{h}|d1\ldots d_{K},T\right)\;d\left(d_{h}\right)\;,$$ (9)

and apply Eq (5) to the second term in the integrand,

$$p\left(d_{0}|d_{1}\ldots d_{K},w_{1}\ldots w_{K},T\right)=\int p\left(d_{0}|d_{h},w_{0}\right)\prod_{k=1}^{K}{\left(\frac{p(d_{h}|d_{k},\tau_{k})}{p(d_{h})}\right)}^{w_{k}}d(d_{h}).$$ (10)

We now make the very reasonable assumption that the evolution of the distance between pairs of atoms manifests diffusive behaviour. This behaviour results if the change in distance can be modelled by many small, independent changes, each change being the consequence of a sequence mutation that will slightly change the protein structure. Concretely, this means the probability of observing a distance *d*_*l*_ after an evolutionary time *τ*_*kl*_, when in the ancestor the distance was *d*_*k*_, is given by

$$p\left(d_{l}|d_{k},\tau_{kl}\right)=N\left(d_{l}|d_{k},\gamma \tau_{kl}\right)\;$$ (11)

with some rate constant *γ*. Note that at time *τ*_*kl*_ = 0 the standard deviation vanishes and the right hand-side becomes equal to the delta functional, as it should. Substituting the conditional probabilities in the integral with these expressions, we see that the integral is over a product of Gaussians and can be solved analytically by the method of completing the square (see Suppl. Material). This results in a Gaussian distribution which is shown in the Supporting Information to be equivalent to the tree $T^{\prime }$ with transformed weights $w_{k}^{\prime }$ given by Eq (8).

For simplicity, we use the UPGMA algorithm [19] to construct the initial tree T. The distances are computed as dist(*t*_*k*_, *t*_*l*_) = −log(TMscore_pred_(*t*_*k*_, *t*_*l*_)), where TMscore_pred_ is the TMscore [20] predicted by a neural network similar to the one in the next subsection (Supplemental Fig. S1), but without the experimental resolution as input feature. The tree constructed in this way is subsequently rearranged so that the query *q* is at its root.

Note that by its construction the final tree with star-like topology has the same edge lengths between the query and any template as the real tree. This is important, since the restraint function for template *t*_*k*_ from the mixture density network depends on the similarity between *q* and *t*_*k*_. In order for the new star-like tree to be equivalent to the real one, it has to represent the same pairwise *q* − *t*_*k*_ similarities as the real tree.

### Template selection

#### Single template selection

HHsearch ranks templates by the probability *P*_hom_ for the template to be homologous to the query protein. To pick the template best-suited for homology modeling, we trained a simple neural network with three hidden nodes (Supplemental Fig. S1) on the training set (see Results). The network predicts the TMscore [20] of the model built with the query-template alignment, given various alignment features described in Table B in S1 Text. The idea is similar to [21], who proposed a neural network (NN) for picking the first template. We tried several feature combinations and, similar to previous work described in [22], found that the following features yielded the best results: HHsearch raw score, secondary structure similarity score divided by query length, expected number of correctly aligned target residues divided by query length, resolution of template structure in Angstroms. For each query, we picked the protein with highest predicted TMscore among all proteins found by HHsearch as the first template.

#### Multiple template selection

Picking the right set of templates for homology modeling is a difficult problem. The main beneficial effect of adding more templates is to increase the number of residues for which distance restraints can be generated [7]. However, picking too many templates can decrease the model quality because, as we discussed in the context of how Modeller’s restraints work, even a single bad template that gives rise to wrong distance restraints can severely distort the resulting 3D model.

To our knowledge, no theoretically well founded strategy for multi-template protein homology modeling has been developed so far, which contrasts with its widespread use in virtually every successful prediction pipeline. Contrary to single template selection, picking further templates is fundamentally complicated by complex dependencies between all selected structures. Current methods are therefore based on heuristics [23–25]. Some methods [26, 27] build a set of models based on several different template lists and then post-select a final model according to some quality measure [28].

As a simple baseline approach to multiple template selection, we employ the network of the previous section to select the first template. Further templates are added if 1) their predicted TMscore is at least 90% of the first template, 2) they are structurally similar to the first template (TMalign score > 0.7) and 3) all selected templates are structurally similar to each other (pairwise TMalign score > 0.8).

Next, we propose here a heuristic method which aims to optimise the trade-off between increasing the query sequence coverage and decreasing the restraint quality of already covered residues due to adding more diverged templates with less reliable alignments.

**Fig 6:**
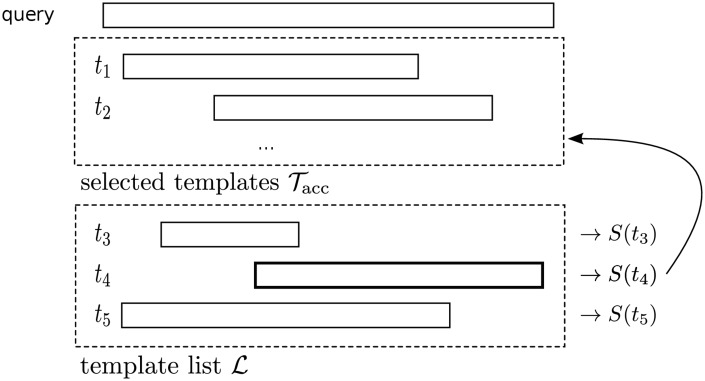
Selection of multiple templates. $T_{\text{acc}}$ is the set of accepted templates, L is the set of template candidates. For each template in L, its score is calculated according to Eq (14) and the template with the highest score (*t*_4_) is added to $T_{\text{acc}}$. This process is iterated until there is no more template with a positive score, or $T_{\text{acc}}$ contains more than 8 templates.

We select the set of templates from among the top 100 found by HHsearch in the following way (Fig 6). The first template *t*_1_ is selected by the neural network that predicts the TMscore. For each template in the template list L (lower dashed box in the figure) a score *S*(*t*) in (see Eq 14) is (re)calculated that rewards a high coverage while penalising the addition of templates whose alignment quality is worse than that of already selected templates. The template with highest score (*t*_4_ in Fig 6) is added to the selected set if its score is still positive. The process is iterated until no template is left in L that has a positive score.

To calculate the score *S*(*t*) (see Eq 14 below), we first define the local quality score,

$$s\left(i,t\right)=P_{hom}\left(t\right)\;p\left(i◇i^{\prime }|q,t\right),$$ (12)

which is simply the product of the probability *P*_hom_ that template *t* is homologous to *q* times the probability *p*(*i* ◇ *i*′∣*q*, *t*) that residue *i* from *q* is homologous (i.e. correctly aligned) to residue *i*′ in *t*. The latter probability is estimated by HHalign and HHsearch by a Forward-Backward algorithm. The local improvement (or impairment) of *s*(*i*, *t*) with respect to the best local score *s*(*i*, *t*′) among already selected templates $t^{\prime }\in T_{\text{acc}}$ is

$$\Delta s\left(i,t\right)=s\left(i,t\right)-\max_{t^{\prime }\in T_{\text{acc}}}\left\{s\left(i,t^{\prime }\right)\right\}.$$ (13)

To weight the positive values more strongly than the negative ones, we apply the exponential function to Δ*s*(*i*, *t*), subtract a per-residue threshold *β* and sum over all aligned residue pairs (*i*, *i*′) in the alignment *A*(*q*, *t*) between *q* and *t*:

$$S\left(t\right)=\sum_{\left(i,i^{\prime }\right)\in A\left(q,t\right)}\left[\;e^{\alpha \Delta s(i,t)}-\beta \;\right].$$ (14)

The parameters *α* and *β* influence the degree of non-linearity and greediness of the selection, respectively. They were optimised with a simple grid search on the optimisation set as explained in the Results section.

## Results

### Benchmark sets

We filtered the sequences from the PDB database of protein structures (May 2010) down to 20% and 70% maximum pairwise sequence identity and a minimal pairwise E-Value of 0.1 (using scripts pdb2fasta.pl and pdbfilter.pl in the HHsuite package v2.0.16). For all sequences in the resulting pdb20 and pdb70 databases, we built multiple sequence alignments (MSAs) with our sensitive, iterative sequence search tool HHblits (v2.0.16) that is based on the pairwise alignment of profile hidden Markov models (Hmms) [15]. We used standard HHblits parameters with three search iterations against the uniprot20 database to get sufficiently diverse MSAs that are well suited to detect even remotely homologous proteins. The query sequences were picked from among the pdb20, and the template database was obtained from the pdb70 as explained below.

We extracted three disjoint query sets from the pdb20, a test, a training and an optimization set, with 1000, 1000, and 500 proteins, respectively. To achieve a good balance of easier and more challenging queries for modeling, we aimed to obtain the same distribution of query-template sequence identities as for the 108 queries in the CASP7 experiment shown in Supplemental Fig. S2 (which is similar to the distribution in CASP11, see Fig. S2). We computed the total amount of queries needed in each sequence identity bin (0%–5%, 5%–10%, …, 95%–100%). We then randomly picked query sequences from the pdb20 without replacement. For each picked query, we searched for possible templates in the pdb70 database and found the template most structurally similar to *q* according to TMalign (excluding the query itself) and recorded the sequence identity given by TMalign. *q* was then put into one of the three sets if the sequence identity bin for that set was not yet filled up. Otherwise, *q* was rejected. Finally, for each of the three query sets we constructed a template set by removing the sequences in the query set from the pdb70.

We then searched with each query sequence *q* in one of the three sets through the corresponding template database using HHsearch, a slower and slightly more sensitive version of HHblits, resulting in a list tlist(*q*) of potential templates.

### Distance restraints

#### Mixture density network

As training data for the mixture density networks for two-component distance restraints, we used the 3D models generated with single templates picked by the neural network. The alignment features for the network were again parsed from the HHsearch results. We fitted distributions of log(*d*) with the mixture of two Gaussians. Modeller includes four different classes of distances depending on the atom types involved: between two *C*_*α*_ atoms (*C*_*α*_–*C*_*α*_), N-O atoms, side chain—main chain and side chain—side chain. We generated four sets of training data with 3 million training cases for *C*_*α*_–*C*_*α*_ and N–O pairs, 1 million for SC–MC and 300*k* for SC–SC. Optimizing the log-likelihood of the mixture density network was done by conjugate gradient ascent until convergence was reached. Bad local optima were avoided by picking the run with maximum likelihood from among 50 random initializations.

#### Two-component distance restraints

**Fig 7:**
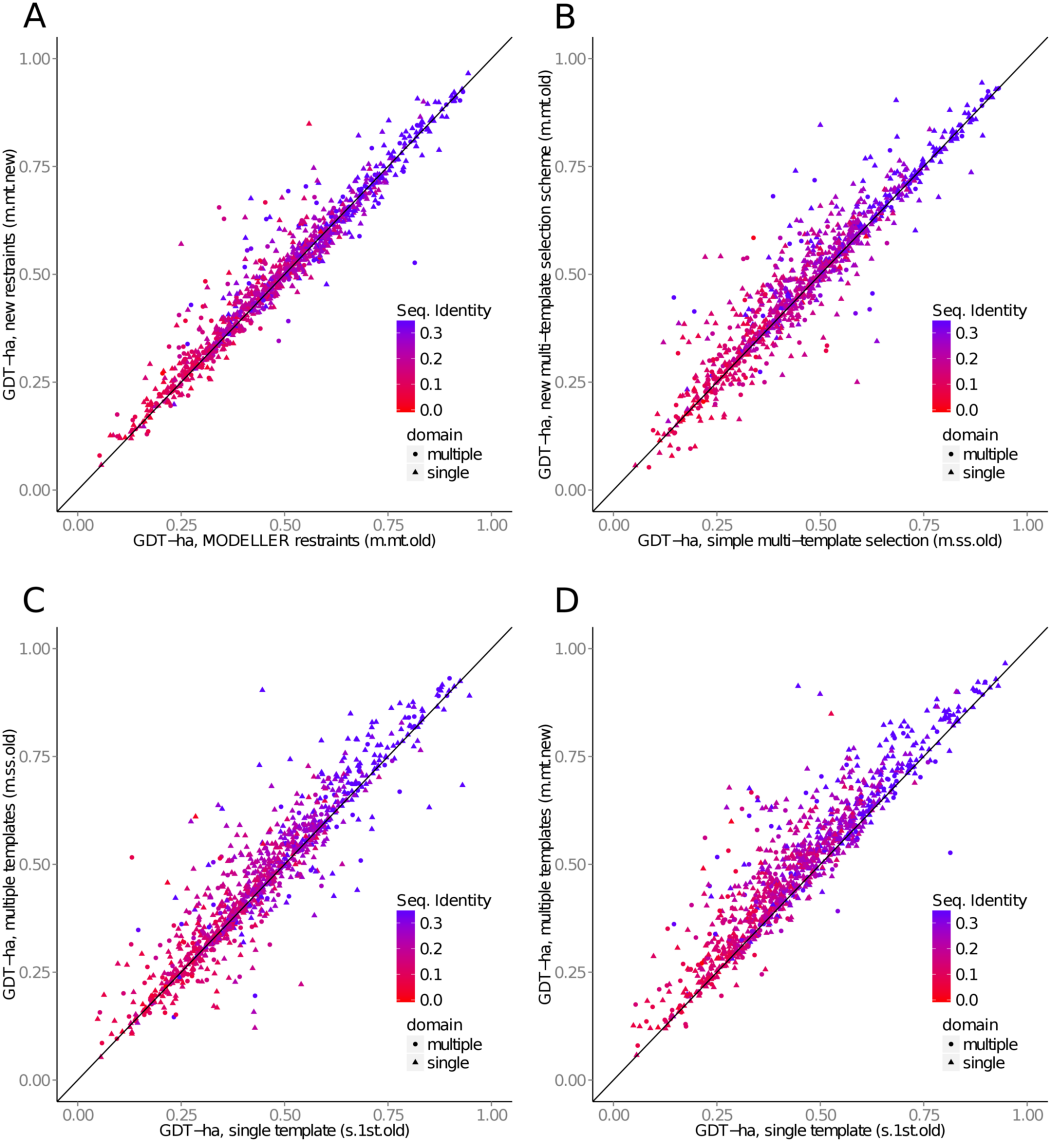
(A) Our two-component mixture restraints improve GDT-ha model quality over Modeller’s default restraints in multi-template modelling by 2.5% on average. (B) Our multi-template selection strategy improves GDT-ha scores over the simple multi-template selection strategy by 3.9% on average. (C) Multi-template modeling improves GDT-ha scores over single-template modelling (using Modeller restraints) by 4.3% on average. (D) Overall improvements through new restraints, template weights, and the new multiple template selection over the baseline, single-template version (s.1st.old in Table 1) is 11.1%.

We replaced all of Modeller’s template based distance restraints with our new two-component Gaussian mixture restraints. The optimization schedule was kept unchanged. The new restraints improved single-template modeling by 0.8% from a GDT-ha model quality score (GDT-ha is a high accuracy version of GDT-ts, see [29]) of 0.447 to 0.450, even though they were developed with multiple template modeling in mind. We then investigated the influence of replacing the new restraints when using our new multi-template selection strategy. We obtained an improvement of the average GDT-ha score over the 1000 queries in the test set by 2.5%, from 0.480 to 0.492 (Table 1 and Fig 7A), which is highly significant according to a paired t-test (P-value: < 2.2 × 10^−16^).

**Table 1:** Average model quality scores for different variations of template selection strategies and restraints used with Modeller on a test set of 1000 single- and multi-domain proteins in the pdb20 database. The GDC-all score is similar to GDT-ha but also includes side-chain atoms in its assessment. Percent improvements are with respect to the first line. P-values are calculated based on a paired t-test with respect to the GDT-ha score in the previous line.

Method				GDT-ha	P-value	GDC-all
Name	Templates	Template selection	Restraints			
s.1st.old	Single	first hit	Modeller	0.443 (+0%)	-	51.9 (+0%)
s.NN.old	Single	neural network	Modeller	0.447 (+0.9%)	1.5E-6	52.4 (+1.0%)
s.NN.new	Single	neural network	new	0.450 (+1.5%)	8E-6	52.8 (+1.7%)
m.ss.old	Multiple	simple selection	Modeller	0.462 (+4.3%)	1E-10	53.5 (+3.1%)
m.mt.old	Multiple	new multi-template	Modeller	0.480 (+8.4%)	2E-16	55.1 (+6.2%)
m.mt.new	Multiple	new multi-template	new	0.492 (+11.1%)	2E-16	56.3 (+8.5%)

### Template selection

#### Single template neural network

We selected the first template based on the query-template alignment features produced by HHsearch using the neural network with three hidden nodes shown in Fig. S1 (see Methods). To train the network, we built 3D models with Modeller (version 9.10) for each query in the training set and each of the maximal 10 best-ranked HHsearch hits in tlist(*q*) as templates. This yielded 9212 models (since some queries had less than 10 database matches), whose model quality we evaluated using TMscore. To learn the network parameters we ran a standard back-propagation procedure. In order to avoid local optima, training was started from several random initializations, which all turned out to optimise to a similar likelihood on the training-set. The correlation between the network predictions and the true TMscore values was 0.89. Compared to selecting the first hit in the HHsearch results list for single template modeling, the neural network-based template selection led to a 0.9% increase of the average GDT-ha from 0.443 to 0.447 (Table 1).

#### Multiple template selection

Choosing multiple templates increases both the coverage and the probability to detect a correct template. However, a higher number of templates leads to accumulation of noise and wrong templates which decreases the model quality. As described in the Methods section, our template selection heuristic has two parameters, *α* and *β*. They were optimized on a grid (*α*, *β*) ∈ {0.9, 0.95, 1, 1.05, 1.1} ⊗ {0.8, 0.9, 1, 1.1, 1.2} using all sequences in the optimization set as queries. For each parameter combination, templates were selected according to the score in formula (14). The alignment features between the query and all templates then served as input for Modeller and 3*D* structures were generated. We found *α* = 0.95 and *β* = 1 to maximize the cumulative TMscore of all models.

The new multi-template selection strategy picked on average 4.6 templates per query (Supplemental Fig. S3), which resulted in a mean coverage of 94% of the query residues (i.e. 94% of query residues where aligned to at least one template residue). The new selection strategy leads to an improvement of 3.9% from an average GDT-ha score of 0.462 to 0.480 (Fig 7B) compared to the following baseline selection strategy: We sorted all templates with respect to their predicted TMscore given by the single template neural network. The first template in this list is always selected and up to 10 templates along this ranking are chosen as long as their predicted TMscore is at least 90% of the very first one (Table 1).

Compared to the single template modeling approach, the improvement of multiple-template modeling without any further refinements (using the simple selection strategy and Modeller restraints) was 4.3%, from average GDT-ha 0.443 to 0.462 (Table 1 and Fig 7C). The total improvement from the baseline, single template modelling to the most refined multi-template modelling strategy sums up to 11.1% (Fig 7D, fist and last line in Table 1).

Note that the improvement in modelling quality of multiple vs. single template modelling does not show a dependence on GDT-HA scores or sequence identities. In other words, difficult targets profit to the same degree as simpler targets from using multiple templates. This is consistent with the observation that both for single- and multi-domain targets, the average number of selected templates was similar across the entire range of sequence identities tested, from 0% to 80% (Supplemental Fig. S3).

### Evaluation on cores

Most model quality assessment scores, such as the GDT-ha, do not penalize incorrect regions and thus reward adding more templates to increase the fraction of the query structure for which restraints can be derived. [30] assessed the effect of using a single or multiple templates on model quality and concluded that most of the gains are due to increased coverage of query residues by template residues. We wanted to discriminate between improvements in model quality due simply to increased coverage and improvements owed to reducing statistical noise by increasing the number of distance restraints on “core residues”, conveniently defined here as residues covered by the alignment to the first, top-ranked template. We remove all non-core residues in the input alignment to Modeller. In that way, distance constraints can only be generated on cores. Then we evaluate the resulting models on core residues only and we compare the GDT-has with the general case.

Table 2 shows that, first, using multiple templates leads to a clear improvement over single templates both in the core regions and overall. This shows that the effect of adding further templates to the first selected template does indeed improve model quality to a similar extent in the core and non-core regions. Similarly, the improvements due to our new two-component restraints are of the same order in the core regions (+2.0%) as overall (+2.5%), leading to a similar conclusion, that the new restraints improve the model to the same extent in the core and non-core regions.

**Table 2:** Multi-template homology modeling and the new restraints improve models within core regions independent of increased query sequence coverage. Mean GDT-has on query protein core regions, defined as the residues that are covered by the first template. Percent improvement with respect to the previous line.

Templates	Selection	Restraints	GDT-ha score overall	GDT-ha score cores only
Single	first hit	Modeller	0.443 (-)	0.464 (-)
Multiple	new multi-template	Modeller	0.480 (+8.4%)	0.504 (+8.6%)
Multiple	new multi-template	new	0.492 (+2.5%)	0.514 (+2.0%)

### Robustness with respect to wrong restraints

Our probabilistic multi-template modeling approach should have the advantage over the Modeller restraints of being more robust towards wrong restraints, because the new distance restraints become flat when log *d* deviates strongly from log *d*_*t*_, i.e., when the restraint cannot be satisfied at all. Therefore, completely wrong restraints practically get ignored in the new approach. Note that this was not a design target of our method but it is simply a consequence of a correct statistical treatment. To test our hypothesis on the robustness of the new restraints, we modified the template selection as follows.

For each query in the test set, we constructed three different template sets (Table 3). The three sets contained two good templates each, and 0, 1 or 2 bad templates, respectively. The good templates were the top two templates according to the TMscores predicted by the neural network in Fig. S1 that also attained a true TMscore of > 0.5. The bad templates were the lowest ranked templates with a true TMscore < 0.3. The average model quality obtained with these three selections are shown in Table 3. As expected, the models built with the new restraints proved to be considerably more robust than the models built with the standard Modeller pipeline.

**Table 3:** The probabilistic multi-template modeling approach is less negatively affected by bad templates. Mean GDT-ha scores of 1000 models built with templates sets containing 0, 1 and 2 bad templates (TMscore < 0.3) along with two good templates (TMscore > 0.5).

Good templates	Bad templates	Modeller restraints	New restraints
2	0	0.474 (-)	0.480 (-)
2	1	0.466 (−1.7%)	0.475 (−1.0%)
2	2	0.458 (−3.4%)	0.471 (−1.9%)

### CASP assessment

CASP (Critical Assessment of Structure Prediction) is a community wide, double-blind experiment that takes place every second year to objectively test the performance of various predictors. HHpred regularly participates in the server based structure prediction category competing with 70–80 other servers. For CASP9 and CASP10, we integrated all methods described above into the HHpred pipeline.

**Fig 8:**
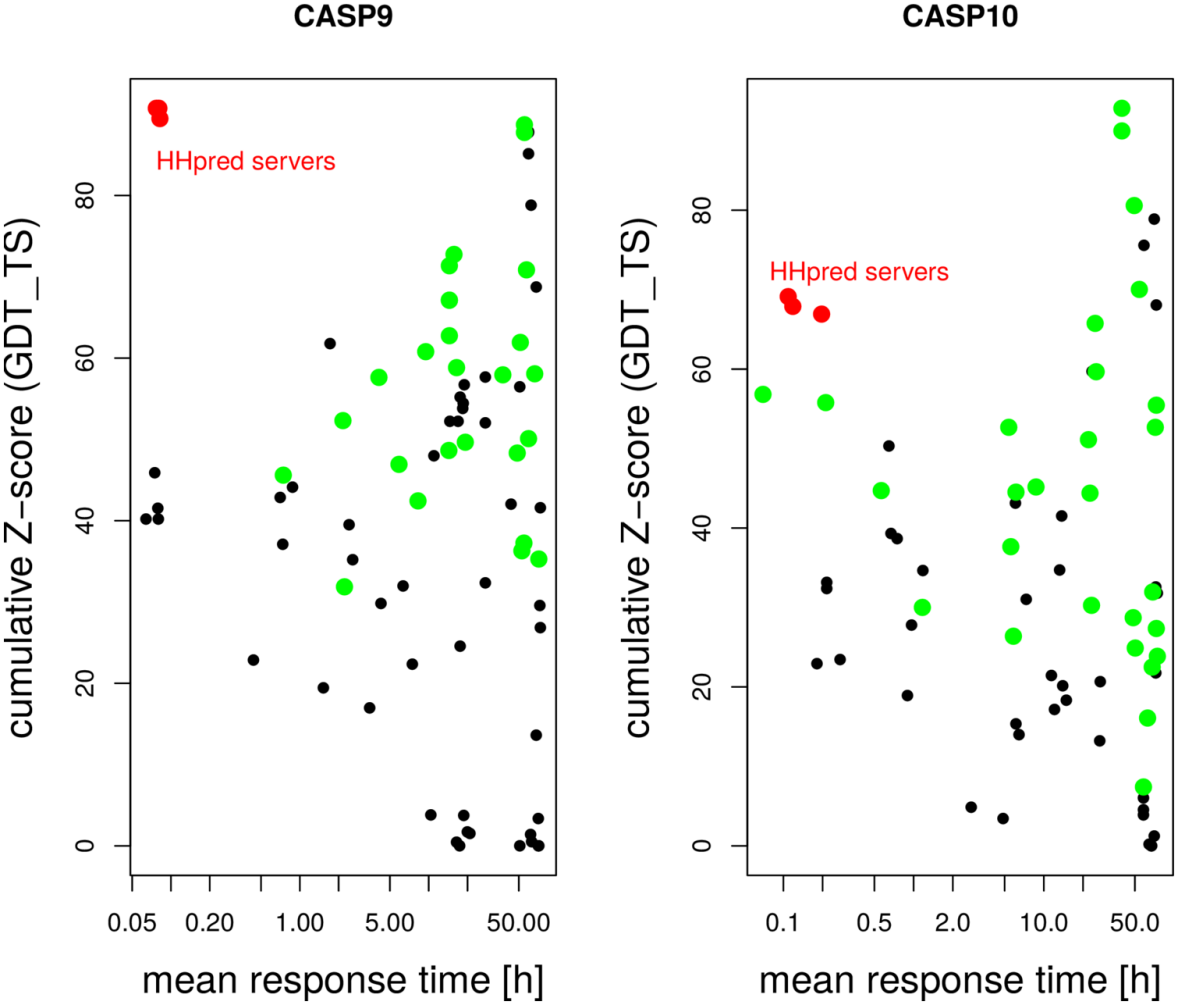
Cumulative Z-score of all server predictions in the template-based modeling category of the CASP9 and CASP10 community-wide assessment of techniques for protein structure prediction [1, 3]. HHpred servers are red, other servers using our HHsuite software are shown in green.

Depending on whether there existed a suitable template in the databases, all queries are subdivided into two categories: template based (TBM) and free (FM). Due to the ever increasing database sizes, most of the queries are TBM (121 vs. 26 in CASP9 and 111 vs. 15 in CASP10). As Fig 8 shows, for TBM HHpred is among the most accurate servers (top 1 in CASP9 and top 7 in CASP10 according to the official CASP ranking—all three servers differ only in minor technical details, see [31, 32]). At the same time HHpred is faster by a factor of ∼ 350 compared with the other leading groups. Fig 8 summarizes the official results in the TBM category from two community-wide assessments of techniques for protein structure prediction, CASP9 (121 query proteins) and CASP10 (111 query proteins) [1, 3]. The values used in the figure were downloaded from the official CASP website (http://predictioncenter.org/). For detailed results, see Supporting Information. When replacing the new restraints with Modeller’s default restraints for the CASP10 set on the same selection of templates, the gdtts-score decreased by 3%.

When considering HHpred’s performance in CASP9 and CASP10, note that assessors filtered out targets that will be too simple to predict by eliminating targets for which a high-confidence homologous template could be found using HHsearch. This procedure thus selectively biases the targets at the detriment of HHpred by eliminating targets that would be easy for HHpred to predict.

## Discussion

Protein structure prediction is a mature field, in which the best methods differ only by a few percent in performance according to recent CASP benchmarks. Even so, great progress has been made in the last 10 to 15 years in template-based protein structure prediction, fuelled by advances in techniques for remote homology detection and alignment [6] and techniques for model quality assessment [3]. In contrast, most successful servers in CASP employ Modeller to build their 3D homology models, a software whose core has changed very little since its publication 22 years ago. This speaks to the enormous success of Modeller’s statistical approach to homology modeling. In this study we have shown how to generalize the statistical approach by taking account of alignment errors and treating restraints from multiple templates in a probabilistically satisfactory way.

These theoretical insights have led to improvements in average model quality (around 6.5%) that are somewhat smaller than what we expected initially. In hindsight, Modeller’s heuristic to derive multi-template restraints works surprisingly well. Also, since Modeller’s internal workings (e.g. the stochastic optimization) are optimized together with its own restraints, it might well be possible to improve on the presented results by specifically optimizing Modeller’s model building procedure with our new restraints. We note, however, that an average model score improvement of 4.4% (m.ss.old versus m.mt.new in GDT-ts, see Table A in S1 Text) corresponds to the difference in GDT-ts scores between the 3rd best and 14th best server in CASP10 [5]. This is a considerable success in particular because our theoretical approach is quite general and can be transferred to other homology modelling methods and to the up-and-coming field of modeling large protein complexes from heterogeneous experimental data [33].

We noted during our tests that the positive impact of the new restraints on model quality is strongest when evaluated with the strictest score, GDT-ha, as compared to the less strict GDT-ts or TMscore (Table A in S1 Text). Here, strictness refers to how severely already small deviations of the model from the true structure are penalized. This observation shows that the improvements of our new restraints are to a substantial degree in the high-precision regime, i.e., below 1 Å, by further improving regions of the model that are already fairly well modeled. Since the best-modeled regions are expected to largely coincide with the highly conserved and hence functionally most important parts of the protein, we expect the new restraints to have the strongest impact on the functionally most important regions of the model.

We are convinced of the power of probability theory in describing quantitative phenomena under uncertainty. Modeller is an excellent case in point. An interesting idea is to carry the probabilistic view further by probabilistically integrating structural and sequence information. All approaches so far start from a fixed query-template alignment (or from a set of alternative alignments) and try to find the 3D model that is best compatible with the alignment. To allow information from the 3D modelling to be fed back to the alignment stage and vice versa, it seems promising to explore the joint posterior probability distribution of alignment and 3D structure. One way to do this would be by Markov Chain Monte Carlo Gibbs sampling of the alignment and the model structure from appropriate conditional distributions.

## Supporting Information

S1 Text Contains supplemental figures (single template neural network, sequence identity distributions), tables (model quality scores, alignment features, CASP results) and methods (details of template weighting). (PDF)

S1 Fasta Training, optimization and test set. These correspond to the supplemental files S2_training_set, S2_test_set.fasta and S2_optimization_set.fasta, respectively.
(ZIP)
