# Supplementary material for: Automatic Prediction of Protein 3D Structures by Probabilistic Multi-template Homology Modeling
Source: PLoS Comput Biol. 2015 Oct 23;11(10):e1004343. doi: 10.1371/journal.pcbi.1004343 (PMC4619893; doi:10.1371/journal.pcbi.1004343)
Supplement: S1 Text — (PDF) [file pcbi.1004343.s001.pdf]

Supplements: Probabilistic multi-template protein homology modeling

A. Meier, J. Soeding

1 Single template neural network

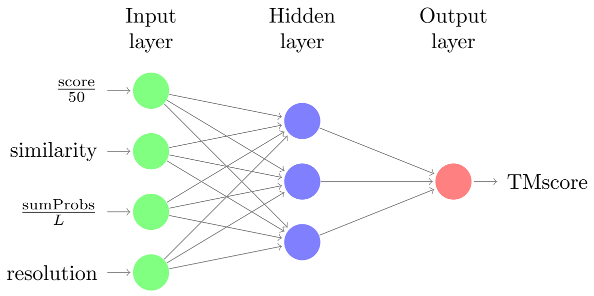

Supplemental Figure 1: Neural network for ranking templates. As input it gets four alignment features and it outputs a predicted TMscore which is then used to find the best template.

2 Sequence identity distribution

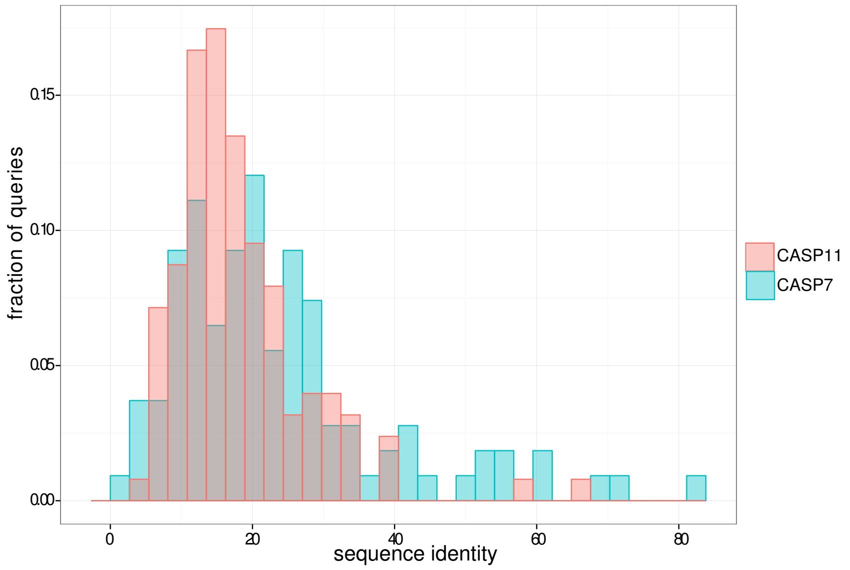

Supplemental Figure 2: Sequence identity histograms of CASP7 and CASP11 targets. The CASP7 distribution serves as a reference for the benchmark, training and optimization set, the CASP11 distribution is for comparison. In both cases around 80% of the targets have a sequence identity between 5 and 30%.

3 Multiple templates

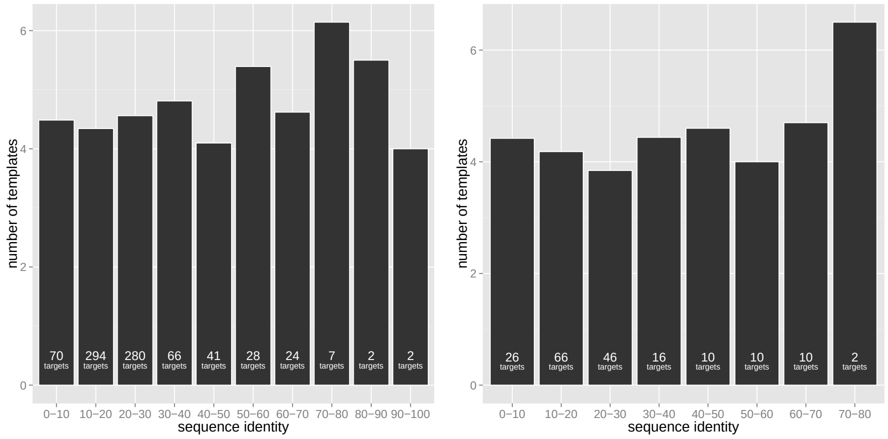

(a) single domain (b) multiple domains

Supplemental Figure 3: Mean number of templates selected by probability based method as a function of sequence identity of the query q to the best template in its list $tlist_q$.

4 Additional scores

Method									
Name	Templates	Selection	Restraints	GDT-ha	P-value	GDT-TS	GDC-all	TM-score	CAD[1]
s.1st.old	Single	first `hhsearch` hit	Modeller	0.443	-	0.614	51.88	0.684	0.572
s.NN.old	Single	neural net	Modeller	0.447	1.47e-6	0.621	52.38	0.694	0.571
s.NN.new	Single	neural net	new	0.450	0.0008	0.623	52.79	0.698	0.575
m.ss.old	Multiple	simple selection	Modeller	0.462	1.43e-10	0.632	53.51	0.703	0.575
m.mt.old	Multiple	new multi-template	Modeller	0.480	2.2e-16	0.648	55.08	0.714	0.574
m.mt.new	Multiple	new multi-template	new	**0.492**	2.2e-16	**0.660**	**56.30**	**0.725**	**0.583**

Supplemental Table A: Extended version of Table 1 in the main text containing additional scores (GDT-TS, GDC-all, TM-score, CAD). Average scores for various variations of template selection strategies and restraints used with Modeller on a test set of 1000 single and multi domain proteins in the pdb20 database. P-values (wrt GDT-ha) are calculated based on a two-sided paired t-test with respect to the previous line. According to Figure 2 in [1], the CAD score has a more limited range than the other scores, which might explain its low variance within our benchmark.

5 Alignment features

Supplemental Table B: Alignment features: these features describe query-template alignments in quantitative numbers and help to rate the alignment quality. All are calculated either within HHsearch or based on its output.

Feature	Description
Probability	The Probability of a template to be a true positive. For the probability of being a true positive, the secondary structure score in column SS is taken into account, together with the raw score. True positives are defined to be either globally homologous or they are at least homologous in parts, and thereby locally similar in structure. More precisely, the latter criterion demands that the MAXSUB score between query and hit is at least 0.1. In almost all cases the structural similarity will we be due to a global OR LOCAL homology between query and template.
Sum of posteriors	The sum of all posterior probabilities along the alignment A between query q and template t, i.e. $$\text{SoP} = \sum_{(q_i, t_{i'}) \in A} P(q_i \diamond t_{i'} \vert q, t),$$ where $P(q_i \diamond t_{i'} \vert q, t)$ is the posterior probability of residues i in the query and i' in the template are correctly aligned. Since SoP is heavily length dependent, it is usually divided by the query length $\vert q \vert$.
Raw score	The raw score is what comes out of the (Viterbi) HMM-HMM alignment excluding the secondary structure score. Informally speaking, it is the sum over the similarities of aligned profile columns minus the gap penalties.
Posterior of two pairs	We denote the posterior probability for two pairs of residues (i, i') and (j, j'); $i < j$, $i' < j'$ being aligned correctly as: $$P(M_i^q \diamond M_{i'}^t,\ M_j^q \diamond M_{j'}^t \vert q, t) \quad (1)$$ Due to the computational complexity to accurately calculate (1), we approximate it as: $$P(M_i^q \diamond M_{i'}^t,\ M_j^q \diamond M_{j'}^t \vert q, t) \approx \begin{cases} \min\{P(M_i^q \diamond M_{i'}^t), P(M_j^q \diamond M_{j'}^t \vert q, t)\}, \\ \quad \text{if } j - i = j' - i' \\ P(M_i^q \diamond M_{i'}^t) \cdot P(M_j^q \diamond M_{j'}^t \vert q, t), \\ \quad \text{otherwise} \end{cases} \quad (2)$$ I.e. when (i, i') and (j, j') lie on the same diagonal in the dynamic programming matrix, we use the minimum, and otherwise the positions are assumed to be independent and can be multiplied.
SS score	The secondary structure score. This score tells you how well the PSIPRED-predicted (3-state) or actual DSSP-determined (8-state) secondary structure sequences agree with each other. PSIPRED confidence values are used in the scoring, low confidences getting less statistical weight.
Similarity	The Similarity is the arithmetic mean of the substitution scores between the aligned residue pairs from the query and template.

6 Determination of template weights

In the following, we describe how to calculate template weights given a tree that specifies the evolutionary relations between a query and templates and among all templates. We are interested in the distance between a given pair residues in the query, d_0, given the corresponding template distances $d_1, \ldots, d_K$ and the pairwise alignments of the query with each template. We assign each template a weight w_k, $k = 1, \ldots, K$ ($w_0 = 1$ for the query) that represents the influence of the leaf on the query. We model the distribution of d_0 given by the left tree $\mathcal{T}$ in Figure 4 as follows (see also in the main text):

$$\frac{P(d_0|d_1,\ldots,d_K,w_1,\ldots,w_K,\mathcal{T})}{P(d_0)} = \int \frac{P(d_0|d_h,w_0)}{P(d_0)} P(d_h|d_1,\ldots,d_K,w_1,\ldots,w_K,\mathcal{T})\mathrm{d}(d_h) \tag{3}$$

where

$$\begin{aligned} P(d_h|d_1,\ldots,d_K,w_1,\ldots,w_K,\mathcal{T}) &= \frac{P(d_1,\ldots,d_K|d_h,w_1,\ldots,w_K,\mathcal{T})P(d_h)}{P(d_h|d_1,\ldots,d_K)} \\ &= \left(\frac{P(d_1|d_h,\tau_1)}{P(d_1)}\right)^{w_1} \cdot \ldots \cdot \left(\frac{P(d_K|d_h,\tau_K)}{P(d_K)}\right)^{w_K} P(d_h) \end{aligned} \tag{4}$$

As mentioned in the main text, we assume a diffusive behaviour with variance proportional to time τ_k (as given by the tree edge lengths):

$$\frac{P(d_k|d_h,\tau_k)}{P(d_k)} \underset{d_h,d_k}{\propto} \exp\left(-\frac{(d_h-d_k)^2}{\tau_k}\right) \quad \forall\, k = 0,\ldots,K. \tag{5}$$

The times τ_k are given by the UPGMA clustering. Then (3) becomes with respect to d_0:

$$\frac{P(d_0|d_1,\ldots,d_K,w_1,\ldots,w_K,\mathcal{T})}{P(d_0)} \propto \int \exp\left(-\sum_{k=0}^{K}\frac{w_k}{\tau_k}(d_h-d_k)^2\right)\mathrm{d}(d_h) \tag{6}$$

The argument in the exponent can be transformed into a quadratic expression of d:

$$-\underbrace{\left(\sum_{k=0}^{K}\frac{w_k}{\tau_k}\right)}_{\frac{1}{\tau_{\min}}} d_h^2 + 2\left(\sum_{k=0}^{K}\frac{w_k}{\tau_k}d_k\right)d_h - \sum_{k=0}^{K}\frac{w_k}{\tau_k}d_k^2 = -\frac{1}{\tau_{\min}}\left(d_h^2 - 2\left(\sum_{k=0}^{K}u_k d_k\right)d_h + \sum_{k=0}^{K}u_k d_k^2\right) \tag{7}$$

where we defined:

$$u_k := \frac{w_k/\tau_k}{\sum_{k'=0}^{K}\frac{w_{k'}}{\tau_{k'}}} = \frac{\tau_{\min}}{\tau_k}w_k \tag{8}$$

Completing the square in (7) gives:

$$-\frac{1}{\tau_{\min}}\left(\left(d_h - \sum_{k=0}^{K}u_k d_k\right)^2 - \left(\sum_{k=0}^{K}u_k d_k\right)^2 + \sum_{k=0}^{K}u_k d_k^2\right) \tag{9}$$

When integrating over d_h (eq 6), the factor:

$$\exp\left[-\frac{1}{\tau_{\min}}\left(\sum_{k=0}^{K}u_k d_k^2 - \left(\sum_{k=0}^{K}u_k d_k\right)^2\right)\right] \tag{10}$$

can be pulled out of the integral (since it is independent of d_h); the integral itself yields a constant that does not depend on d_0. Therefore:

$$\frac{P(d_0|d_1,\ldots,d_K,w_1,\ldots,w_K,\mathcal{T})}{P(d_0)} \underset{d_0}{\propto} \exp\left(-\frac{1}{\tau_{\min}}\left(\sum_{k=0}^{K}u_k d_k^2 - \left(\sum_{k=0}^{K}u_k d_k\right)^2\right)\right) \tag{11}$$

Now, we want to find new weights w'_k such that (see Figure 4; this step describes the transition from the left tree to the right one):

$$\frac{P(d_0|d_1,\dots,d_K,w_1,\dots,w_K,\mathcal{T})}{P(d_0)} \overset{!}{\underset{d_0}{\propto}} \exp\left(-\sum_{k=1}^{K}\frac{w'_k}{\tau_0+\tau_k}(d_0-d_k)^2\right) \tag{12}$$

Here, the last step introduced new weights w'_k so that the template distances $d_1,\dots,d_K$ become directly dependent on the query distance d_0. Now, an expression for w'_k, $k=1,\dots,K$ must be found. We equate the arguments of the exp functions according to eqs. (11) and (12):

$$\sum_{k=0}^{K} u_k d_k^2 - \left(\sum_{k=0}^{K} u_k d_k\right)^2 = \tau_{\min}\sum_{k=1}^{K}\frac{w'_k}{\tau_0+\tau_k}(d_0-d_k)^2 + \mathrm{const}(d_0) \tag{13}$$

We collect terms with equal powers of d_0:

$$(u_0-u_0^2)d_0^2 - \left(2u_0\sum_{k=1}^{K}u_k d_k\right)d_0 = \left(\tau_{\min}\sum_{k=1}^{K}\frac{w'_k}{\tau_0+\tau_k}\right)d_0^2 - \left(2\tau_{\min}\sum_{k=1}^{K}\frac{w'_k}{\tau_0+\tau_k}d_k\right)d_0 + \mathrm{const}(d_0) \tag{14}$$

Equating coefficients in eq. (14) leads to:

$$\begin{aligned} u_0(1-u_0) &= \tau_{\min}\sum_{k=1}^{K}\frac{w'_k}{\tau_0+\tau_k} \\ \sum_{k=1}^{K}u_k d_k &= \frac{\tau_{\min}}{u_0}\sum_{k=1}^{K}\frac{w'_k}{\tau_0+\tau_k}d_k \end{aligned} \tag{15}$$

We demand that the w'_k are proportional to the old weights w_k. Then the scaling factors result from equating all individual summands in eq. (15):

$$u_k = \frac{\tau_{\min}w'_k}{u_0(\tau_0+\tau_k)} \quad \forall k=1,\dots,K \tag{16}$$

and solving for w'_k gives:

$$w'_k = \frac{\frac{1}{\tau_0}+\frac{1}{\tau_k}}{\frac{1}{\tau_{\min}}}w_k \tag{17}$$

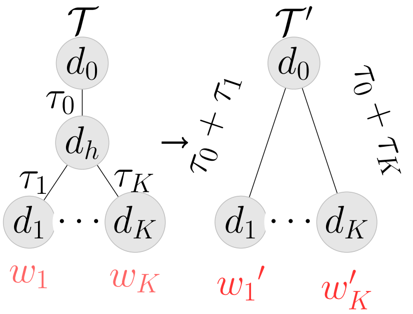

Supplemental Figure 4: Illustration of restructuring a given tree $\mathcal{T}$ with an hidden node d_h (left) into one where $d_1,\dots,d_K$ directly depend on d_0 (right). This is done by integrating over d_h and finding appropriate weights $w'_1,\dots,w'_K$ so that both trees describe the same distribution. $t_1,\dots t_K$ correspond to distances obtained by UPGMA clustering.

We apply formula (17) iteratively starting from a UPGMA tree rooted at the query until there is only the root node d_0 and directly connected template nodes $d_1,\dots,d_K$, i.e. no more hidden nodes (see also main text). The final w_k^{final}, $k=1,\dots,K$ are used as template weights.

7 Additional CASP9 and CASP10 results

Supplemental Table C: CASP9 overall results: official CASP9 results for all servers for both TBM and FM targets. The table is sorted with respect to the sum Z-score column. Time is given in mean minutes per target.

rank	server	dom	sum Z-score	avg Z-score	avg GDT-TS	time [min]
1	QUARK	147	115.788	0.788	62.675	3358.736
2	Zhang-Server	147	113.242	0.77	62.765	3347.378
3	RaptorX-MSA	147	103.27	0.703	61.774	3586.239
4	RaptorX	147	103.01	0.701	61.731	3587.406
5	RaptorX-Boost	147	99.845	0.679	61.453	3587.241
6	HHpredB	147	93.104	0.633	59.528	4.334
7	HHpredA	147	93.104	0.633	59.528	4.405
8	HHpredC	147	91.821	0.625	59.361	4.398
9	Seok-server	147	89.542	0.609	60.158	3735.85
10	MULTICOM-CLUSTER	147	88.944	0.605	59.987	1030.446
11	BAKER-ROSETTA	145	87.24	0.602	58.768	3518.86
12	MULTICOM-REFINE	147	86.441	0.588	59.519	1030.697
13	MULTICOM-NOVEL	147	82.825	0.563	59.371	1030.873
14	gws	145	82.645	0.57	58.931	4147.591
15	chunk-TASSER	147	82.609	0.562	58.846	3220.107
16	Phyre2	147	78.792	0.536	58.823	989.234
17	MULTICOM-CONSTRUCT	147	76.446	0.52	58.703	1030.728
18	pro-sp3-TASSER	147	75.358	0.513	58.117	3227.174
19	MUFOLD-Server	147	68.676	0.467	56.26	3991.966
20	FAMSD	147	68.669	0.467	57.295	624.882
21	ZHOU-SPARKS-X	147	68.644	0.467	57.727	105.152
22	JiangAssembly	146	68.195	0.467	56.999	1197.921
23	Pcomb	142	64.774	0.456	57.809	1651.211
24	PconsD	147	64.422	0.438	56.483	2454.316
25	JiangTHREADER	146	64.206	0.44	57.096	1214.558
26	SAM-T08-server	140	63.211	0.452	56.193	1102.817
27	PconsM	143	63.133	0.441	57.598	1065.89
28	Bilab-ENABLE	147	62.233	0.423	54.818	1106.28
29	IntFOLD-TS	147	58.906	0.401	55.732	246.478
30	prdos2	145	58.14	0.401	55.682	3690.435
31	GSmetaserver	137	57.376	0.419	57.234	1184.296
32	BioSerf	147	56.438	0.384	53.403	85.008
33	Pcons	139	56.068	0.403	58.032	129.937
34	ProQ2	141	55.475	0.393	56.205	1648.542
35	CLEF-Server	147	55.266	0.376	55.543	928.274
36	chuo-fams	147	54.884	0.373	55.173	876.997
37	FALCON-SWIFT	147	54.55	0.371	55.223	863.763
38	circle	134	53.942	0.403	57.968	2912.41
39	MUFOLD-MD	145	53.183	0.367	51.588	3248.714
40	3D-JIGSAWV4-0	144	51.932	0.361	54.954	299.138
41	FFAS03n	145	51.361	0.354	53.883	4.362
42	ProfileCRF	147	51.054	0.347	54.144	660.07
43	MidwayFoldingServer	139	47.239	0.34	52.137	4417.612
44	FFAS03	139	46.974	0.338	54.553	3.63
45	PconsR	142	46.515	0.328	55.279	2602.051
46	3D-JIGSAWV4-5	140	46.238	0.33	54.617	472.421
47	Atome2CBS	138	45.723	0.331	53.508	43.482
48	PRECORS	140	45.272	0.323	52.051	3364.469
49	Distill	147	45.181	0.307	53.183	139.991
50	MUSTER	146	45.051	0.309	53.06	43.5
51	FFAS03ss	140	42.915	0.307	53.905	4.664
52	FFAS03a	141	42.615	0.302	54.028	4.843

53	Wolfson-serv	145	37.863	0.261	50.628	39.966
54	LOOPPAustin	130	36.934	0.284	55.738	159.893
55	MUSICSserver	141	35.939	0.255	44.402	4312.718
56	ProQ	125	34.124	0.273	54.247	1644.804
57	PROTAGORAS	122	33.636	0.276	53.326	132.509
58	panther	116	32.811	0.283	52.586	405.392
59	LMUserver	130	31.22	0.24	50.316	4382.261
60	SAM-T02-server	127	30.227	0.238	53.518	261.543
61	YASARA	76	27.587	0.363	65.559	4393.479
62	SAM-T06-server	137	26.809	0.196	49.451	1048.062
63	Ma-OPUS-server	147	25.127	0.171	45.441	442.9
64	FUGUEKM	131	23.52	0.18	50.935	26.127
65	Pushchino	123	19.839	0.161	43.831	208.166
66	m4t2009	61	19.551	0.321	65.033	93.125
67	MUSICS-2S	115	13.623	0.118	42.488	4114.478
68	RaptorX-FM	21	11.026	0.525	33.908	3601.972
69	LenServer	126	8.831	0.07	24.987	3724.417
70	RBO-PROTEUS	143	8.356	0.058	27.327	1045.136
71	rehtnap	110	4.311	0.039	39.967	588.546
72	STAT-PROTEUS	127	3.527	0.028	23.841	1255.585
73	Yangkdd	125	2.203	0.018	27.353	1235.588
74	ConStruct	109	1.647	0.015	20.172	1031.664
75	BHAGEERATH	147	1.49	0.01	18.689	3761.989
76	PLATO	111	1.422	0.013	19.647	1075.657
77	schenk-torda	31	0	0	21.913	3082.077
78	Fortmannserver	11	0	0	11.537	4310.783
79	PHAISTOSserver	1	0	0	18.502	4265.172

Supplemental Table D: CASP10 overall results: official CASP10 results for all servers for both TBM and FM targets. The table is sorted with respect to the sum Z-score column. Time is given in mean minutes per target.

**rank**	**server**	**dom**	**sum Z-score**	**avg Z-score**	**avg GDT-TS**	**time [min]**
1	Zhang-Server	126	111.874	0.888	60.601	2457.093
2	QUARK	126	105.531	0.838	60.204	2462.948
3	BAKER-ROSETTA	126	87.787	0.697	57.542	2977.735
4	RaptorX-ZY	126	85.964	0.682	58.43	4250.788
5	RaptorX	126	82.911	0.658	58.055	3606.894
6	TASSER-VMT	126	82.016	0.651	57.382	3307.054
7	PMS	126	78.113	0.62	57.559	4378.698
8	HHpred-thread	124	77.339	0.624	58.402	11.766
9	HHpredA	126	76.748	0.609	57.563	6.486
10	HHpredAQ	126	75.904	0.602	57.295	6.635
11	PconsM	126	73.806	0.586	56.42	1492.026
12	Pcons-net	125	72.226	0.578	55.072	1550.339
13	chunk-TASSER	126	69.35	0.55	56.323	1615.115
14	MULTICOM-REFINE	125	64.94	0.52	55.848	4.192
15	MULTICOM-NOVEL	119	62.581	0.526	56.532	12.892
16	MULTICOM-CLUSTER	126	62.192	0.494	55.92	325.825
17	Mufold-MD	126	59.984	0.476	54.661	4323.788
18	MUFOLD-Server	127	59.031	0.465	55.057	4385.074
19	MULTICOM-CONSTRUCT	121	56.558	0.467	53.665	39.283
20	Phyre2A	126	53.903	0.428	54.419	1964.666
21	ZHOU-SPARKS-X	126	50.963	0.404	53.774	34.305
22	FALCON-TOPO	126	50.211	0.398	53.918	338.068
23	FALCON-TOPO-X	126	49.897	0.396	53.429	338.186
24	Seok-server	126	48.788	0.387	54.303	524.859
25	PconsD	125	46.754	0.374	52.787	1465.628

26	SAM-T08-server	113	46.004	0.407	55.662	816.348
27	Distill	126	41.665	0.331	53.195	47.894
28	hGen3D	126	41.652	0.331	51.579	40.483
29	NewSerf	126	38.303	0.304	51.341	70.537
30	MUFoldCRF	122	37.536	0.308	49.915	4359.16
31	IntFOLD2	126	37.447	0.297	51.66	347.904
32	samcha-server	113	36.259	0.321	47.878	797.276
33	3D-JIGSAWV5-0	120	35.572	0.296	52.623	435.009
34	Bilab-ENABLE	126	33.981	0.27	49.68	1392.564
35	chuo-fams-server	126	33.567	0.266	52.385	4152.381
36	FFAS03c	125	32.699	0.262	51.309	13.063
37	slbio	118	32.017	0.271	51.111	4440.532
38	FFAS03mt	112	31.849	0.284	54.496	12.874
39	Distillroll	126	31.391	0.249	50.196	58.01
40	Atome2CBS	111	29.897	0.269	52.219	69.422
41	MATRIX	114	29.256	0.257	51.868	2992.605
42	chuo-repack-server	126	29.161	0.231	50.55	4403.425
43	STRINGS	111	29.006	0.261	52.524	3046.083
44	JiangServer	126	27.694	0.22	47.726	684.899
45	IntFOLD	126	27.456	0.218	49.152	350.171
46	FRESSserver	126	25.429	0.202	49.235	4461.935
47	AOBA-server	124	25.163	0.203	49.258	4083.524
48	FFAS03hj	112	24.636	0.22	53.387	16.303
49	JiangFold	126	24.28	0.193	46.255	901.773
50	JiangThreader	126	23.226	0.184	45.935	53.331
51	YASARA	77	23.22	0.302	63.858	4346.388
52	FFAS03	111	22.475	0.202	53.557	10.829
53	UGACSBL	109	21.864	0.201	53.247	1632.667
54	GSmetaserver	74	20.817	0.281	56.069	834.386
55	SAM-T06-server	112	19.728	0.176	49.373	387.024
56	BhageerathH	125	19.132	0.153	43.315	3751.958
57	sysimm	62	17.655	0.285	55.967	642.096
58	RBO-MBS	121	16.504	0.136	24.44	3494.306
59	PROTAGORAS	100	15.66	0.157	53.795	366.478
60	RaptorX-Roll	20	14.757	0.738	25.77	213.993
61	RBO-i-MBS	121	14.243	0.118	24.634	3494.168
62	panther	94	13.211	0.141	49.135	1635.295
63	RBO-MBS-BB	121	9.749	0.081	25.484	3492.71
64	RBO-i-MBS-BB	121	9.633	0.08	24.639	3493.005
65	HOMER	93	5.569	0.06	41.815	166.603
66	Lenserver	40	2.268	0.057	27.084	4232.178
67	confuzz3d	46	0.676	0.015	22.026	3993.645
68	confuzzGS	58	0.388	0.007	27.818	4025.334
69	Bhageerath	5	0.185	0.037	40.766	3854.73

References

[1] Olechnovic,K. *et al.* (2013) CAD-score: a new contact area difference-based function for evaluation of protein structural models, *Proteins*, **81**, 149–62.
